# The impact of social exclusion vs. inclusion on subjective and hormonal reactions in females and males^☆^

**DOI:** 10.1016/j.psyneuen.2013.07.021

**Published:** 2013-12

**Authors:** E.M. Seidel, G. Silani, H. Metzler, H. Thaler, C. Lamm, R.C. Gur, I. Kryspin-Exner, U. Habel, B. Derntl

**Affiliations:** a Department of Health, Development and Psychological Intervention, Faculty of Psychology, University of Vienna, Vienna, Austria; b Social, Cognitive and Affective Neuroscience Unit, Department of Basic Psychological Research and Research Methods, Faculty of Psychology, University of Vienna, Vienna, Austria; c Cognitive Neuroscience Sector, International School for Advanced Studies, SISSA-ISAS, Trieste, Italy; d Neuropsychiatry Division, Department of Psychiatry, University of Pennsylvania, Philadelphia, USA; e Department of Psychiatry, Psychotherapy and Psychosomatics, RWTH Aachen University, Aachen, Germany

**Keywords:** Gender, Progesterone, Testosterone, Cortisol, Social exclusion, Cyberball

## Abstract

**Background:**

The experience of social exclusion represents an extremely aversive and threatening situation in daily life. The present study examined the impact of social exclusion compared to inclusion on steroid hormone concentrations as well as on subjective affect ratings.

**Methods:**

Eighty subjects (40 females) participated in two independent behavioral experiments. They engaged in a computerized ball tossing game in which they ostensibly played with two other players who deliberately excluded or included them, respectively. Hormone samples as well as mood ratings were taken before and after the game.

**Results:**

Social exclusion led to a decrease in positive mood ratings and increased anger ratings. In contrast, social inclusion did not affect positive mood ratings, but decreased sadness ratings. Both conditions did not affect cortisol levels. Testosterone significantly decreased after being excluded in both genders, and increased after inclusion, but only in males. Interestingly, progesterone showed an increase after both conditions only in females.

**Discussion:**

Our results suggest that social exclusion does not trigger a classical stress response but gender-specific changes in sex hormone levels. The testosterone decrease after being excluded in both genders, as well as the increase after inclusion in males can be interpreted within the framework of the biosocial status hypothesis. The progesterone increase might reflect a generalized affiliative response during social interaction in females.

## 1 Introduction

The experience of social exclusion represents an extremely aversive situation in daily life. Social exclusion can threaten fundamental human needs: belonging, self-esteem or control (Williams, 2007). Benenson et al. (2011) proposed that social exclusion is a form of non-directed aggression that is particularly salient to females. Women are socialized to create more intimate, close relationships while males are encouraged to develop a more independent relationship style and focus on self-autonomy (Cyranowski et al., 2000). As a result, women's self-concept is more strongly based on connectedness to others (Cross and Madson, 1997). It has further been hypothesized that females suffer more than males from negative social situations by worrying and attributing these experiences to a lack of personal competence (Rose and Rudolph, 2006). In the present study, we aimed at investigating gender differences in subjective and hormonal responses to social exclusion vs. inclusion.

Previous studies have documented that experimentally induced social exclusion triggers a number of affective responses. A meta-analysis of experimental social exclusion studies using different paradigms (Blackhart et al., 2009) showed larger effect sizes for mood changes as a function of exclusion when samples had a higher proportion of females. Studies using a virtual ball tossing game, the Cyberball paradigm (Williams et al., 2000), found that Cyberball exclusion in samples with a female majority was associated with higher anger (Zadro et al., 2004) and depression ratings (Zoller et al., 2010). Weik et al. (2010) reported increased anger after Cyberball exclusion in both genders, but increased depression ratings only in females. Notably, studies using samples with a higher proportion of male participants also reported mood decreases after Cyberball exclusion (Wesselmann et al., 2012), but others with a sole male sample did not report increases in anger (Geniole et al., 2011). With respect to subjectively experienced distress, however, no previous Cyberball study with mixed samples (e.g., Boyes and French, 2009; Hawkley et al., 2011; Kelly et al., 2012) has explicitly assessed gender differences. Therefore, the first aim of the present study was to examine whether social exclusion has more negative effects on females compared to males on a subjective level. Due to lacking prior experimental evidence, we expected stronger negative responses in females based on theoretical considerations only.

Apart from subjective distress ratings, social exclusion may also affect the release of the major stress hormone, cortisol. Blackhart et al. (2007) showed elevated cortisol levels after subjects have been told that none of their previous interaction partners wanted to work with them. Using the Yale Interpersonal Stressor Task, Stroud et al. (2002) reported stronger cortisol and blood pressure increase in females, but no gender differences in self-reported distress, when being excluded and rejected by two interaction partners that connected well with each other. Notably, studies using the Cyberball paradigm did not observe cortisol increases in the exclusion groups, either in pure male (Geniole et al., 2011), pure female (Zoller et al., 2010), nor in a (rather small) mixed sample (Zwolinski, 2012). Given previous null findings in cortisol response when applying the Cyberball paradigm as an experimental manipulation of social exclusion, we wanted to further investigate whether social exclusion triggers cortisol release in females and males differently. We assumed that if social exclusion does impact cortisol, this effect would be stronger in females based on pioneering findings by Stroud et al. (2002).

However, social exclusion might not only result in gender differences in cortisol responses, but could also affect the release of major sex hormones, such as testosterone and progesterone. Building on the biosocial status hypothesis (Mazur and Booth, 1998), a recent review by Eisenegger et al. (2011) postulated that testosterone plays a role in a broader picture involving power and dominance motives, such as the search for and the maintenance of social status. There are only two previous Cyberball studies investigating effects on testosterone. One study found that increases of testosterone in both sexes correlated with anger changes (Peterson and Harmon-Jones, 2012) but did not report significant testosterone changes from before to after the task. The other likewise did not find a significant change in testosterone responses in a male sample in either exclusion or inclusion (Geniole et al., 2011). Despite limited evidence on testosterone responses to Cyberball exclusion, we expected social exclusion to result in testosterone decrease, related to a loss in social status, as outlined by Eisenegger et al. (2011).

There is limited evidence on the involvement of progesterone in social motivation. Initial studies suggest a positive correlation between progesterone and implicit affiliation motivation in male and female subjects independent of cycle phase (Schultheiss et al., 2003; Wirth and Schultheiss, 2006). Accordingly, Brown et al. (2009) observed increased progesterone after a closeness condition compared to a neutral condition in a female sample. In addition, Wirth (2011) proposed that progesterone release may be especially responsive to social rejection. With regard to social exclusion, Maner et al. (2010) observed that in socially anxious subjects remembering experiences of social exclusion led to a decrease in progesterone. In contrast, subjects with no social anxiety showed an increase in progesterone levels. In a second experimental manipulation subjects were told that a previous online interaction partner did not want to meet them, which resulted in progesterone increases in subjects with high rejection sensitivity. Given the scarce previous evidence on progesterone responses to social interaction manipulations, the present study aimed at further exploring these effects. Pioneering results by Maner et al. (2010) suggest progesterone increase in response to social exclusion. However, based on previous results by Brown et al. (2009) one might also assume that social inclusion increases progesterone levels.

Taken together, no previous study investigated the impact of gender on subjective as well as hormonal responses to social exclusion vs. inclusion in a large gender-balanced sample controlling for menstrual cycle phase in female subjects. Based on experimental results in female majority samples (e.g., Weik et al., 2010; Zadro et al., 2004; Zoller et al., 2010) and theories suggesting that social exclusion may be more relevant for females (e.g., Benenson et al., 2011; Rose and Rudolph, 2006), we expected that gender is of critical relevance in this context. This is the first study using virtual social exclusion, such as the Cyberball paradigm, that tested for gender differences in subjective ratings (e.g., Boyes and French, 2009; Hawkley et al., 2011; Kelly et al., 2012). There is some evidence of cortisol increase in response to real-life exclusion paradigms (Blackhart et al., 2007; Stroud et al., 2002); however, previous Cyberball studies could not find effects on cortisol (Geniole et al., 2011; Zoller et al., 2010; Zwolinski, 2012). There are only two studies (Geniole et al., 2011; Peterson and Harmon-Jones, 2012) examining the impact of Cyberball exclusion on testosterone, yielding no significant changes. Moreover, there is only scarce evidence on the impact of social interaction manipulations on progesterone suggesting an association with affiliative motivation (Brown et al., 2009; Maner et al., 2010).

## 2 Methods

### 2.1 Participants

Eighty non-smoking Vienna University students (40 females) were randomly assigned to an exclusion or an inclusion experiment. Forty students (20 females) participated in the exclusion and forty students (20 females) participated in the inclusion experiment. Psychology students were excluded because they might have knowledge or suspicion about the deception involved in the Cyberball task. We investigated students to obtain homogenous samples concerning age (exclusion sample: mean = 24.78 years (SD = 3.02); inclusion sample: 24.18 years (SD = 3.6); *t*(78) = 0.804, *p* = 0.424) and intelligence (exclusion sample: IQ = 105.9 (SD = 10.9); inclusion sample 102.3 (SD = 9.5); *t*(78) = 1.760, *p* = 0.082). Furthermore, the two samples did not differ in social anxiety (*t*(76) = 0.677, *p* = 0.501). Also, male and female participants did not differ in age (*t*(78) = 1.419, *p* = 0.160), intelligence (*t*(78) = 0.189, *p* = 0.850) or social anxiety (*t*(76) = 1.757, *p* = 0.083). In order to control for menstrual cycle effects, all females were tested in the mid-luteal phase (in a 28-day cycle: between days 18 and 23). Based on previous findings using agentic stressors (Kajantie and Phillips, 2006; Lustyk et al., 2010) one can expect females in the luteal phase to show a comparable cortisol response to males. None of the female subjects was taking oral contraceptives and no participant was taking any other type of hormonal medication. Participants were recruited via advertisements posted at the Medical University of Vienna and the University of Vienna, Austria. Written informed consent was obtained. The study was approved by the local Institutional Review Board and subjects were treated according to the Declaration of Helsinki (1964) regarding the treatment of human research participants. All subjects received € 10 for their participation.

### 2.2 Task

**Figure 1:**
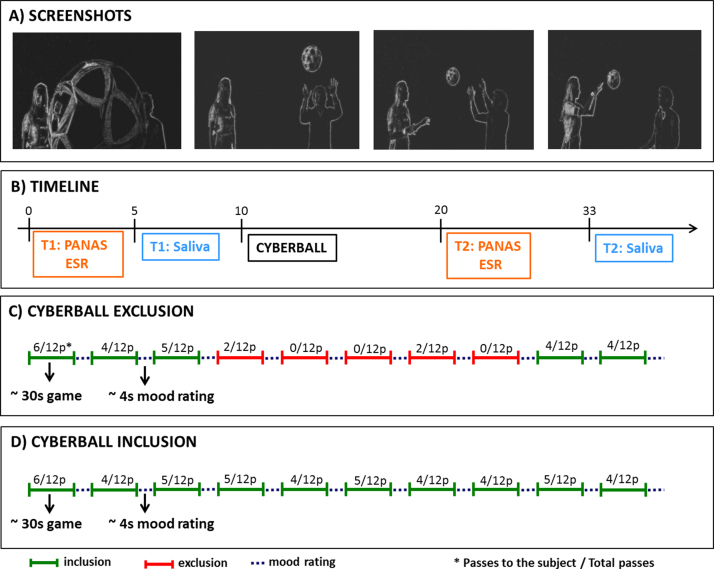
Experimental setup: visualization of the procedure including screenshots of the visual interface with the two other players (Panel A), the timeline of PANAS and ESR ratings as well as saliva samples (Panel B), block structure of the exclusion (Panel C) and the inclusion (Panel D) task.

We applied a modified version of the Cyberball paradigm (Eisenberger et al., 2003; Novembre et al., submitted for publication). Participants engaged in a virtual ball tossing game with what they believed to be two other players sitting in other laboratories in the same building. The game consisted of 10 separate blocks with 12 passes each, and the other players and their ball throws were conveyed to subjects by means of pre-recorded video clips which showed stylized players whose gender was recognizable. In reality, there were no other players; participants were playing with a pre-set computer program and were given a cover story to ensure that they believed the other players were real. During the *exclusion experiment*, the first three blocks were “inclusion” blocks, in which the participant received at least one-third of the passes (see Fig. 1). This procedure was used to induce the experience of being part of the game in the beginning of the experiment. This experience was subsequently spoiled, as the next five blocks were “exclusion blocks” in which the participant received zero or only two passes. For ethical reasons, the last two blocks were inclusion blocks again. During the *inclusion experiment* the subject received around one-third of the passes in each round. In each experiment, and after each block, subjects rated their current mood from negative to positive on a 9-point scale ranging from −4 to +4.

### 2.3 Saliva samples

Participants were told that the study investigated hormonal responses to social interaction. They were further asked to refrain from consuming alcohol or caffeine, and from engaging in strenuous physical activity or exercise 24 h prior to testing; to refrain from eating or drinking anything (except water) for 2 h prior to testing as cortisol is elevated subsequent to each of these activities. To reduce diurnal hormone variability, we asked all participants to arrive between 2 pm and 6 pm. There was no difference regarding time of testing between samples (inclusion vs. exclusion) (*p* = 0.922) or gender (*p* = 0.922), nor was there an interaction effect (*p* = 0.379). Hormone assessment has been performed by a commercial laboratory (SwissHealthMed, Aying, Germany), which used a conventional approach for assaying hormone levels in saliva. Upon arrival of the samples in the analysis laboratory the samples were frozen at −20 °C at least overnight. To precipitate mucins, samples were thawed and centrifuged at 3000–2000 × *g* for 10 min. Competitive Luminescence Immunoassay kits (LUMI) were used to measure concentrations of hormones (testosterone and progesterone as pg/ml, and cortisol as ng/ml). The LUMI kit is based on the competition principle. These kits have minimal cross-reactivity to other steroid hormones. Measurements were highly reliable (progesterone: intra-assay CV < 7% and inter-assay CV < 19%, testosterone: intra-assay CV < 4% and inter-assay CV < 7%, cortisol: intra-assay CV < 4% and inter-assay CV < 5%). The lower limit of sensitivity of the immunoassay kits was 2.6 pg/mL for progesterone, 1.8 pg/mL for testosterone and 0.003 μg/dL for cortisol.

### 2.4 Procedure

Upon arrival to the laboratory, participants were asked to provide demographic information and to fill in the German version of the Social Interaction Anxiety Scale (SIAS; Mattick et al., 1989). Immediately before and after the virtual ball tossing game participants were asked to fill in a computerized version of the Emotional Self Rating (Schneider et al., 1994) and the PANAS (Watson et al., 1988). Questionnaire items and the virtual ball tossing game were presented on a 15.4 inch laptop monitor (Dell Latitude) using Matlab 7.9.0 (The MathWorks, Inc., Natick, MA) and the Matlab-based toolbox Cogent 2000 developed by the Cogent 2000 team at the FIL and the ICN and Cogent Graphics developed by John Romaya at the LON at the Wellcome Department of Imaging Neuroscience, London, UK. Saliva samples were taken before (T1) and 25 min after (T2) the onset of the ball tossing game, which corresponds to 20 min post exclusion onset in the exclusion experiment. Subjects spitted around 1 ml of saliva in small tubes, which were properly closed and frozen until analysis. The timing of post-stress-collection was chosen based on typical response curves of cortisol (Dickerson and Kemeny, 2004). Less is known about response times of progesterone and testosterone. However, our timing approach is based on the suggestions of other studies indicating that initial changes in progesterone (Maner et al., 2010) and testosterone (Schultheiss et al., 2005) can be determined in saliva after approximately 15 min (Schultheiss et al., 2012). Finally, all subjects completed computerized German versions of two questionnaires (NEO Five-Factor Inventory (NEO-FFI): Costa and McCrae, 1992; Bem Sex Role Inventory (BSRI): Bem, 1981). After filling in all questionnaires, participants were fully debriefed about the experiment.

### 2.5 Statistical analysis

Statistical analyses were performed using SPSS (Statistical Packages for the Social Sciences, Version 18.0, SPSS Inc., USA). Hormone data were analyzed with a gender by experiment ANCOVA with hormone T1 values as the covariate and hormone T2 values as the dependent variable. Given previous evidence of an overlap between salivary progesterone and cortisol (see Wirth et al., 2007), we controlled for this by adding cortisol baseline values as an additional covariate in a supplementary progesterone analysis. Rating data were analyzed with a gender by time by experiment ANOVA with repeated measures. In the case of non-sphericity, Greenhouse-Geisser corrected degrees of freedom and *p*-values are listed. For significant effects, estimates of effect size are reported (partial-eta squared for the ANOVAs and Cohen's *d* for post-hoc *t*-tests). Correlation analyses are performed using the Pearson coefficient, or the Spearman coefficient in case of non-normal data distribution. We correlated all change variables (after – prior to ball game, i.e., T2–T1) of hormone data and subjective distress data with each other and with the BSRI ratings. Multiple comparisons or multiple correlations were Bonferroni corrected.

## 3 Results

### 3.1 Cortisol

The 2 (gender) by 2 (experiment) ANCOVA showed no significant main effects or interactions (all *p*-values ≥0.167).

### 3.2 Testosterone

**Figure 2:**
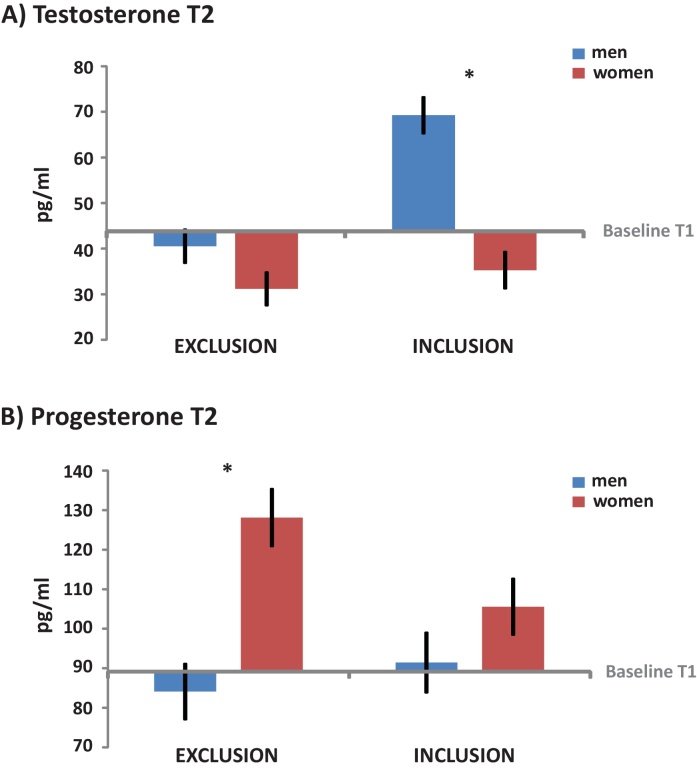
Hormone results (estimated values based on the ANCOVA model controlling for baseline differences): Panel A shows the significant gender by time interaction on testosterone level (in pg/ml) with an increase after social inclusion only in men and a decrease after social exclusion in both genders. Panel B displays the significant gender by time interaction on progesterone levels (in pg/ml) with an increase after social exclusion and inclusion only in women. Significant differences are marked with an asterisk (*p* < 0.001).

The 2 (gender) by 2 (experiment) ANCOVA showed a significant gender difference (*F*(1,75) = 19.108, *p* < 0.001, partial eta^2^ = 0.203) with higher testosterone levels in males compared to females (see Fig. 2). Furthermore, there was a significant main effect of experiment (*F*(1,75) = 22.352, *p* < 0.001, partial eta^2^ = 0.230) with higher testosterone T2 values compared to baseline in the inclusion experiment and lower T2 values in the exclusion experiment. We also obtained a significant gender by experiment interaction (*F*(1,75) = 11.974, *p* = 0.001, partial eta^2^ = 0.138) which is depicted in Fig. 2A.

Follow-up comparisons showed that in the inclusion experiment there was a gender difference in testosterone response (*p* < 0.001) with higher testosterone T2 values compared to baseline in males, whereas in females testosterone T2 values were lower compared to baseline. In the exclusion experiment both genders showed a similar decrease compared to baseline (no gender difference: *p* = 0.10).

### 3.3 Progesterone

The 2 (gender) by 2 (experiment) ANCOVA showed a significant gender difference (*F*(1,74) = 11.139, *p* = 0.001, partial eta^2^ = 0.131) with higher progesterone in females compared to males. Also, a significant gender by experiment interaction (*F*(1,74) = 4.806, *p* = 0.032, partial eta^2^ = 0.061) emerged, see Fig. 2B. There was no main effect of experiment (*p* = 0.269).

Follow-up comparisons showed that there was a gender difference in progesterone response in the exclusion experiment (*p* < 0.001) with females showing a strong increase compared to baseline whereas males did not exhibit any change. For the inclusion experiment there was no gender difference (*p* = 0.214), mean progesterone T2 values were significantly higher compared to baseline. Furthermore, for females there was a difference in progesterone change between experiments (*p* = 0.021) with a more pronounced increase in the exclusion experiment. For males, there was no difference between the two experiments (*p* = 0.455).

Controlling these progesterone analyses for the impact of cortisol by adding cortisol baseline values as an additional covariate, we could not find a significant impact of the covariate (*p* = 0.836). Hence, the above main effects and interactions remained unchanged.

### 3.4 Mood ratings

The 2 (gender) by 2 (experiment) by 2 (time) ANOVA on positive mood ratings on the PANAS showed a significant main effect of time (*F*(1,70) = 9.394, *p* = 0.003, partial eta^2^ = 0.118), as well as a time by experiment interaction (*F*(1,70) = 7.796, *p* = 0.007, partial eta^2^ = 0.100). All other main effects and interactions were non-significant (all *p*-values ≥0.213).

**Figure 3:**
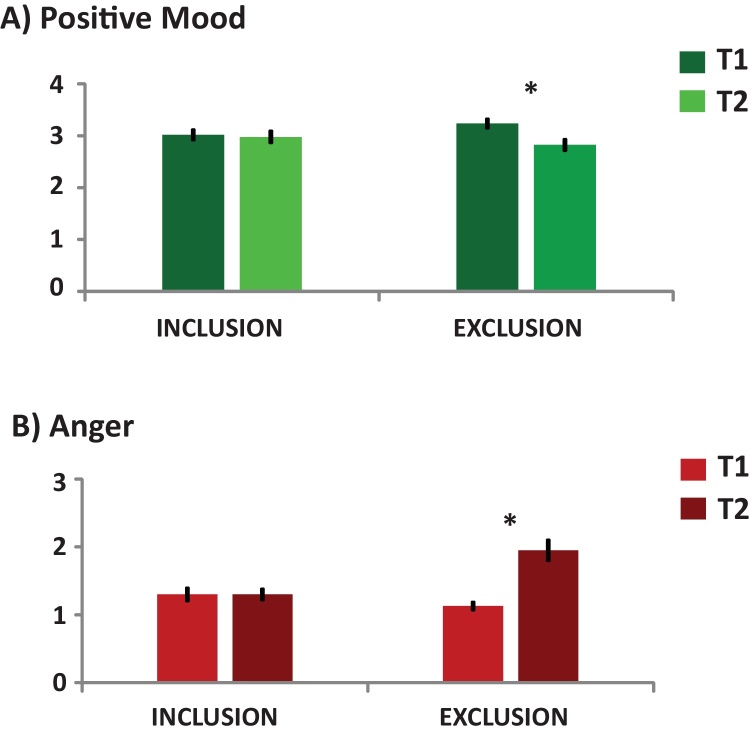
Mood rating results: Panel A displays the significant decrease of mean positive mood ratings after social exclusion. Panel B shows the significant increase in mean anger ratings after social exclusion. Significant differences are marked with an asterisk (*p* ≤ 0.001).

In order to disentangle the time by experiment interaction we performed paired *t*-tests for each experiment. This showed that there was a significant decrease of positive mood ratings in the exclusion experiment (*t*(35) = 3.783), *p* = 0.001, *d* = 0.640), but not in the inclusion experiment (*t*(37) = 0.212, *p* = 0.833), see Fig. 3A.

The 2 (gender) by 2 (experiment) by 2 (time) ANOVA on negative mood ratings on the PANAS revealed a trend for a time by experiment interaction (*F*(1,74) = 3.921, *p* = 0.051, partial eta^2^ = 0.051). All other main effects or interactions remained non-significant (all *p*-values ≥0.599). Paired *t*-tests showed that there was a tendency for a decrease in negative mood ratings (*t*(40) = 1.815, *p* = 0.077, *d* = 0.302) in the inclusion experiment, but not in the exclusion experiment (*t*(37) = 1.161, *p* = 0.253).

A 2 (gender) by 2 (experiment) by 2 (time) ANOVA on anger ratings on the ESR showed a significant main effect of time (*F*(1,74) = 17.367, *p* < 0.001, partial eta^2^ = 0.190) as well as a significant effect of experiment (*F*(1,74) = 4.126, *p* = 0.046, partial eta^2^ = 0.053). Moreover, we observed a significant time by experiment interaction (*F*(1,74) = 17.367, *p* < 0.001, partial eta^2^ = 0.190). All other main effects or interactions remained non-significant (all *p*-values ≥0.110). Post-hoc paired *t*-tests showed that there was a significant increase of anger after the exclusion experiment (*t*(37) = 5.119, *p* < 0.001, *d* = 0.936), but not after the inclusion experiment (*t*(39) = 0, *p* = 1), see Fig. 3B.

A 2 (gender) by 2 (experiment) by 2 (time) ANOVA on sadness ratings on the ESR showed a significant time by experiment interaction (*F*(1,74) = 5.156, *p* = 0.026, partial eta^2^ = 0.065). All other main effects or interactions remained non-significant (all *p*-values ≥0.235). Post-hoc paired *t*-tests showed that only after the inclusion experiment there was a decrease in sad mood (*t*(39) = 2.479, *p* = 0.018, *d* = 0.423) but not after the exclusion experiment (*t*(37) = 1.152, *p* = 0.257).

All other ANOVAs on ESR ratings did not reveal any significant main effects or interactions (all *p*-values ≥0.150).

### 3.5 Correlation analyses

In the exclusion experiment, we observed a strong positive correlation (*r* = 0.856, *p* = 0.002) between mood ratings after each block and the number of passes subjects received in the respective block. This correlation was significant in males (*r* = 0.756, *p* = 0.011) and females (*r* = 0.867, *p* = 0.001). In the inclusion experiment, there was no significant correlation between mood ratings and the number of passes subjects received in the respective block either for the whole group or for males or females separately (all *p*-values ≥0.353).

Moreover in the exclusion experiment, femineity scores of the BSRI positively correlated with changes (T2–T1) in anger ratings (Spearman's *ρ* = 0.487, *p* = 0.002 (corr. 0.008)), as well as changes (T2–T1) in negative mood ratings (*r* = 0.406, *p* = 0.011 (corr. 0.044)). This means that the more feminine our subjects rated themselves the angrier they became and the more negative mood they experienced after social exclusion. Also, there was a significant negative correlation of positive mood change and femineity scores (*r* = −0.363, *p* = 0.030), which did not survive multiple comparison correction (*p*(corr.) = 0.12).

For the inclusion experiment no significant correlations with BSRI occurred (all *p*-values >0.16). Moreover, we did not observe any significant correlations between differences in hormone concentration or subjective ratings prior/after testing with the inclusion experiment and questionnaire data.

There was no significant difference on either masculinity (*t*(78) = 1.601, *p* = 0.113) or femineity (*t*(78) = 0.081, *p* = 0.936) ratings between experiments (exclusion vs. inclusion) or between females and males for each experiment (inclusion: masculinity (*p* = 0.593), femineity (*p* = 0.536); exclusion: masculinity (*p* = 0.190), femineity (*p* = 0.362)).

## 4 Discussion

We investigated gender differences in subjective and hormonal responses to social exclusion in comparison to an inclusion control experiment. There were five principal findings. First, both genders showed a strong emotional reaction after social exclusion, as indicated by changes in self-reported affect. Second, we did not observe significant increases in cortisol, which suggest that Cyberball exclusion does not trigger a classical stress response. Third, testosterone levels decreased after social exclusion in both genders, but increased after inclusion in males only. Fourth, in females progesterone increased after both manipulations, but the increase was significantly stronger after exclusion. Fifth, despite the absence of differences between male and females subjects (biological sex) in subjective distress ratings there was a significant influence of social gender role identification, such that higher femineity was associated with stronger emotional responses to social exclusion. However due to methodological issues, we regard this result as tentative, pending replication in future studies.

Mood and emotion ratings after Cyberball exclusion in our study did not show any gender differences but for the whole sample we observed a significant increase of anger and negative mood in general. This supports previous evidence that the immediate consequences of social exclusion are negative emotional reactions (Gerber and Wheeler, 2009; Kelly et al., 2008; Weik et al., 2010).

Despite showing clear and strong emotional responses, however, we did not observe any change in cortisol values by social exclusion in either males or females in the luteal phase. This is consistent with previous studies using the Cyberball paradigm (males only: Geniole et al. (2011); females only: Zoller et al. (2010); mixed sample: Zwolinski (2012)). However, other social exclusion studies using different manipulations, including interactions with real people, did show cortisol effects in a mixed gender group (Blackhart et al., 2007) as well as in the female sample only (Stroud et al., 2002). Apparently, the virtual-reality nature of the interaction in the Cyberball paradigm in comparison to more immediate real-life interaction and exclusion by real people does not trigger responses of the endocrinological stress axis.

Notably, our social exclusion paradigm had strong effects on testosterone and progesterone. Our results showed a significant testosterone decrease after exclusion in both genders and an increase after inclusion in males. Only two previous studies using the Cyberball paradigm investigated testosterone responses to social exclusion. In a female majority sample Peterson and Harmon-Jones (2012) found that testosterone increase in both sexes strongly correlated with anger changes but did not report significant testosterone changes due to exclusion. Another study also did not observe significant changes in testosterone in a male sample (Geniole et al., 2011). In addition to sample differences (only males vs. female majority), both studies used a task that was considerably shorter than ours (Peterson and Harmon-Jones: 4 min; Geniole et al.: 7 min) and may therefore have been less powerful in inducing testosterone responses. Furthermore, Geniole et al. (2011) used a task with three (male) co-players, wherein exclusion might not be as obvious as when being excluded in a task with only two co-players, as used in our study.

Our results can be interpreted within the framework of the biosocial status theory (Mazur and Booth, 1998) in the sense that social status or related motives, such as power or dominance, are considerably challenged by the exclusion from the ball tossing game in both genders. This conforms to findings that losers in competitive interactions show testosterone decreases whereas winners show increases (e.g., review by Salvador and Costa, 2009). However, when being part of social group, i.e., during the inclusion experiment, the testosterone increase may reflect a boost in social status or feelings of power. It is remarkable that the latter effect is only present in males. This may reflect the higher sensitivity of males to social status motives. Alternatively, male subjects may have mainly passed the ball to the female player. Then, the testosterone increase could reflect the well-known testosterone increases related to reproductive behavior (Archer, 2006, for review). However, the task was not programmed to record ball throw choices. Therefore, this interpretation remains speculative.

Regarding progesterone, we observed an increase in females after both inclusion and exclusion. Pioneering studies suggest a positive correlation between progesterone and implicit affiliation motivation in male and female subjects (Schultheiss et al., 2003; Wirth and Schultheiss, 2006). There is only one previous study showing a modulation of progesterone after social exclusion by personality traits, such that socially anxious subjects showed a decrease whereas subjects high in rejection sensitivity showed an increase in a sample with a majority of male subjects (Maner et al., 2010). However, this study did not control for gender differences. Our result of a progesterone increase in females following both social inclusion and exclusion suggests a rather generalized increase in affiliative motivation after social interaction. Remarkably, males did not show these effects. The more pronounced female-specific progesterone increase after exclusion may reflect that females show a strong desire to re-affiliate after being rejected. One could speculate that after the inclusion task, the increase in progesterone reflected a general affiliative response after social interaction (Brown et al., 2009) or the desire to get to know the other players. In contrast, the increase after the exclusion condition suggests that females would have rather been motivated to compensatory re-affiliate with friends or at least different people than the other players. This could be clarified in future studies applying a detailed post-experimental questionnaire.

Despite the absence of (biological) sex differences in subjective distress ratings, the level of gender role identification had a significant impact on subjective distress ratings after exclusion. More specifically, subjects who rated themselves as more feminine became angrier and showed more negative mood upon social exclusion. This could suggest that social gender compared to biological sex might have a more significant impact on subjective emotional reactions to social exclusion. However, because subjects were administered the BSRI after the Cyberball game, this result awaits replication in future studies applying the questionnaire before the experimental manipulation. Furthermore, this effect was only observed on subjective ratings and we did not observe any correlations with hormonal change data; these results have to be interpreted with caution. The results could mean that exclusion manipulations have a greater effect on feminine subjects (scoring higher on femineity in the BSRI) but they might simply reflect higher levels of emotional expressiveness among those who identify more with a female gender role.

Furthermore, we cannot state that the effects we observed are specific for a social environment, as we did not apply a non-social control game, such as for example passing a ball against a wall. This should be incorporated in future studies using the Cyberball paradigm. Another important additional procedure would be recording physiological measures, such as skin conductance or heart rate, in order to track physiological arousal changes with higher temporal resolution. Moreover, a post-experimental questionnaire on thoughts during the exclusion trials, behavioral measures of ball passing patterns, or a measure of affiliative motivation add further insights into gender differences in responses to social exclusion.

In summary, Cyberball exclusion produced strong subjective and hormonal effects in male and females subjects. We observed increases in anger and negative mood after social exclusion but not after social inclusion. Despite lacking gender differences in subjective distress, hormonal reactions, especially progesterone, showed differences in response to social exclusion. Moreover, our data showed a first hint on correlations between subjective responses to social exclusion and femineity, i.e., social gender role identification, which should be interpreted with caution due to methodological limitations.

## Role of funding source

This study has been supported by the Austrian Science Fund (FWF-PP23533). EMS GS CL received funding from Viennese Science and Technology Fund (WWTF-CS11-016). The FWF and the WWTF had no further role in study design; in the collection, analysis and interpretation of data; in the writing of the report; and in the decision to submit the paper for publication.

## Conflict of interest

All authors declare that they have no conflicts of interest.
